# COVID-19 Recovery Patterns Across Alpha (B.1.1.7) and Delta (B.1.617.2) Variants of SARS-CoV-2

**DOI:** 10.3389/fimmu.2022.812606

**Published:** 2022-02-14

**Authors:** Nitya Kumar, Suha Quadri, Abdulla Ismaeel AlAwadhi, Manaf AlQahtani

**Affiliations:** ^1^ Department of Medicine, Royal College of Surgeons in Ireland –Bahrain, Muharraq, Bahrain; ^2^ Department of Pathology, Bahrain Defense Force Hospital–Royal Medical Services, Riffa, Bahrain

**Keywords:** SARS-CoV-2, COVID-19 vaccine, delta variant, alpha variant, hospital stay length, recovery pattern, B1.617.2, B1.1.7

## Abstract

**Background:**

B.1.1.7 (alpha) and B.1.617.2 (delta) variants of concern for SARS-CoV-2 have been reported to have differential infectivity and pathogenicity. Difference in recovery patterns across these variants and the interaction with vaccination status has not been reported in population-based studies.

**Objective:**

The objective of this research was to study the length of stay and temporal trends in RT-PCR cycle times (Ct) across alpha and delta variants of SARS-CoV-2 between vaccinated and unvaccinated individuals.

**Methods:**

Participants consisted of patients admitted to national COVID-19 treatment facilities if they had a positive RT-PCR test for SARS-CoV-2, and analysis of variants was performed (using whole genome sequencing). Information on vaccination status, age, sex, cycle times (Ct) for four consecutive RT-PCR tests conducted during hospital stay, and total length of hospital stay for each participant were ascertained from electronic medical records.

**Results:**

Patients infected with the delta variant were younger (mean age = 35years vs 39 years for alpha, p<0.001) and had lesser vaccination coverage (54% vs 72% for alpha, p<0.001). RT-PCR Ct values were similar for both variants at the baseline test; however by the fourth test, delta variant patients had significantly lower Ct values (27 vs 29, p=0.05). Length of hospital stay was higher in delta variant patients in vaccinated (3 days vs 2.9 days for alpha variant) as well as in unvaccinated patients (5.2 days vs 4.4 days for alpha variant, p<0.001). Hazards of hospital discharge after adjusting for vaccination status, age, and sex was higher for alpha variant infections (HR=1.2, 95% CI: 1.01–1.41, p=0.029).

**Conclusion:**

Patients infected with the delta variant of SARS-CoV-2 were found to have a slower recovery as indicated by longer length of stay and higher shedding of the virus compared to alpha variant infections, and this trend was consistent in both vaccinated and unvaccinated patients.

## Introduction

The respiratory infection, COVID-19, caused by the novel coronavirus 2 or SARS-CoV-2 that originated in Wuhan, China in December 2019 rapidly devolved into a worldwide pandemic in March of 2020 (1). Despite experiencing differential evolution across geographies and continuing well into 2021, this pandemic also saw unprecedented global initiatives in vaccine development, approval, and implementation efforts that have resulted in a decline in the number and severity of cases (2–5). Several studies have found vaccination to be linked with the reduced number of cases and severity of infection (6–10). However, the widespread disparity in distribution and access of the vaccines (11, 12) as well as the lack of adherence to public health measures to control the spread (13–15) saw unchecked community spread in different geographical locations. This constellation of factors gave rise to multiple mutations in the genetic make-up of the pathogen, resulting in different variants (16).

Some of the SARS-CoV-2 variants have been labeled “variants of concern” including the alpha, beta, gamma, and delta variants (17). The first identified variant was the D614G in March 2020 in China (18). Other notable variants are the beta variant found in South Africa in December 2020 and the gamma variant found in Brazil in January 2021 (19). The B.1.1.7 variant was first detected in September 2020 in Kent, United Kingdom. It was formally termed the “alpha” variant of concern in December 2020 (20).

The B.1.617.2 “delta” variant was first identified in December 2020 during the second wave of COVID-19 in India (20, 21). It garnered global attention due to a high degree of infectivity, morbidity, and mortality than previously witnessed in the pandemic and has become the dominant strain in the US and UK among over ten other countries (8). The variant infects more young patients than previous variants do, and subsequently infection rates have risen in children and adolescents since its spread (22). The viral load for this delta variant is also over a thousand times higher than the original strain (23).

The clinical presentation of the virus has also changed with the variant. Atypical symptoms such as clots, gangrene, mucormycosis, abdominal pain, nausea, diarrhea, hearing loss, myalgia, and arthralgias have been speculated to be caused by delta. Many patients also present afebrile or with mild and nonspecific symptoms (24).

The delta variant has been known to cause breakthrough infections, with some fully vaccinated individuals exhibiting symptomatic infection. Even fully vaccinated infected individuals can spread the virus to others (25). Although vaccines are effective against the variant, multiple studies have found reduced efficacy against the delta variant alone and in comparison to other variants (26). The variant has been found to be two times more communicable than previous variants. Some vaccines and antibody treatments have been less successful against this variant compared to others (27). In addition, morbidity is increased with delta, as it poses twice the risk of hospitalization and need of emergency resources (28).

Besides sporadic reports on the efficacy of various vaccines on the delta variant and preclinical and modeling studies on pathogenicity, the interaction of the effect of vaccination and the pathogenicity of variants of concern, especially the recovery pattern, remains poorly understood in clinical settings. This is partly due to the lack of comprehensive data from infections from different variants in vaccinated and unvaccinated individuals. The Kingdom of Bahrain was one of the very few states to achieve a comprehensive vaccination coverage at the population level (29)) and also saw different variants of concern. Therefore, this study was conducted with the aim of studying the length of hospital stay and pattern of RT-PCR cycle times across vaccinated and unvaccinated patients infected with the SARS-CoV-2 variants B.1.1.7 (here on referred to as the “alpha variant”) and B.1.617.2 (henceforth referred to as the “delta variant”).

## Methods

### Study Population, Patient Selection, and Data Extraction

The study population comprised of patients admitted to COVID-19 treatment facilities under the Bahrain Ministry of Health between 1 January 2021 and 30 May 2021. Patients were included if they were above 18 years of age, had a positive RT-PCR for SARS-CoV-2, were admitted between the above specified dates, and were identified as being infected with either the alpha or delta SARS-CoV-2 variants through whole genome sequencing. Data pertaining to demographic details, COVID-19 test results, vaccination status, and length of hospital stay were extracted from the local electronic medical record (EMR) system, “I-SEHA.” Data were extracted manually from EMR, and all cases were reviewed manually before inclusion into the study.

### Diagnosis of COVID-19

Confirmation of an infection with SARS-CoV-2 was done using a standard reverse transcriptase polymerase chain reaction (RT-PCR) test of nasopharyngeal swab samples. The test was performed using Thermo Fisher Scientific (Waltham, MA) TaqPath 1-Step RT-qPCR Master Mix, CG on the Applied Biosystems (Foster City, CA) 7500 Fast Dx RealTime PCR instrument. This assay targeted the E gene. Once the E gene was detected, the test was confirmed by RdRP and N gene assays. E gene Ct values have been reported in this study. Ct values >40 were considered negative. Positive and negative controls have been included for quality control.

### Ascertainment/Detection of Variants

Whole genome sequencing was used to identify the common variants of concerns using illumina/ARTIC and COVID-Seq protocols. The data were analyzed with the Abiomix platform. Sequencing was undertaken at the national COVID-19 molecular public health laboratory where all the samples get tested. Spike gene target status on PCR was used as a second approach for identifying each variant.

### Outcome Assessment

Recovery pattern among the patients was assessed by estimating the length of hospital stay as well as the longitudinal trend in PCR test cycle time (CT) values indicating the extent of viral shedding, across 4 consecutive PCR tests. As per Bahrain national protocol during the study period, PCR testing was required to be carried out 4 times: test 1 performed at the time of diagnosis (corresponding to day 0), test 2 (performed on day 3), test 3 (performed on day 5), and final test (either on day 10 or earlier if they are clinically stable). Subjects who stayed in the hospital for 10 or more days were tested 4 times. Those who got discharged before day 5 underwent only two tests: diagnosis test and discharge test. Those who got discharged between days 5 and 10 underwent testing three times: diagnosis, discharge, and once on day 3. All patients have minimum two tests—one performed at the time of diagnosis and one performed at the time of recovery or discharge.

### Statistical Analysis

The proportion of vaccination and variants have been described using frequencies and percentages, PCR CT values have been reported as means and standard deviations, and length of hospital stay has been reported as median and interquartile range. Difference in consecutive CT values across SARS-CoV-2 variants was assessed using a mixed model analysis of variance (mixed ANOVA). Length of stay was estimated using Kaplan–Meier analysis. Hazards of hospital discharge adjusted for age and sex were computed using a Cox proportional hazards model. All analyses were performed using STATA 17 (StataCorp. 2020. Stata Statistical Software: Release 17. College Station, TX: StataCorp LLC.)

### Ethics Approval and Declaration

Ethics approval for this study was obtained from the National COVID-19 Research Committee in Bahrain (approval code: CRT-COVID2021-148). All methods and analysis in this study were carried out in compliance with the local guideline and ethical guidelines of the Declaration of Helsinki 1975. All data used in this study were collected as part of regular medical procedures. Given the retrospective nature of the study and the de-identification of patients’ information, the requirement for informed consent was waived by the reviewing body.

### Data Availability Statement

Original data will be made available upon request to the corresponding author.

## Results

The demographic characteristics across the SARS-CoV-2 variants are shown in **Table 1**. Of the study subjects, 636 (44.5%) were infected with the delta variant, and 737 (55.5%) were infected with the alpha variant. Delta variant patients were significantly younger (median age = 35years) than those infected with the alpha variant (median age = 39, p<0.001). Both groups had a more or less comparable sex distribution (**Table 1**). Among the delta variant patients, 353 (54.1%) were vaccinated compared to 573 (72.4%) in the alpha variant group. The mean Ct values for the first two PCR tests were comparable across the groups; however, the mean Ct values for the subsequent two tests seemed significantly lower for the delta variant (p=0.05 for test #4). Our data were collected between January and April of 2021, when the initial vaccines to get approved in the country were Sinopharm, AstraZeneca, Pfizer-BioNTech, and subsequently Sputnik. Among our vaccinated participants, 289 had taken Sinopharm, 21 had taken AstraZeneca, 12 took Pfizer-BioNTech, and 18 were vaccinated with Sputnik (data not shown in the tables). Information on the type of vaccine was not available for the remaining 739 of the subjects who were vaccinated. All subjects who were double vaccinated have been identified as vaccinated in our study, and those who did not receive any dose have been identified as “nonvaccinated.” Individuals who received a single dose or who received booster doses were not included in the study.

**Table 1:** Characteristics of the study subjects.

Characteristics	Alpha variant (n=792)	Delta variant (n=636)	p-value
N (%)	792 (55.5%)	636 (44.5%)	
Age [median, (IQR)]	39 (29, 51)	35 (28, 44)	<0.001
Male [n, (%)]	467 (59.0%)	399 (62.83%)	0.145
Bahraini nationality [n, (%)]	553 (69.8%)	338 (53.1%)	<0.001
Vaccination coverage [n, (%)]	573 (72.4%)	343 (54.1%)	<0.001
PCR test 1 CT value (n=2070) [mean, (SD)]	23.1 (3.8)	22.7 (3.1)	0.13
PCR test 2 CT value (n= 1291) [mean, (SD)]	28.0 (4.0)	27.3 (3.7)	0.24
PCR test 3 CT value (n= 540) [mean, (SD)]	29.2 (4.3)	27.6 (4.2)	0.076
PCR test 4 CT value (n=2070) [mean, (SD)]	29.7 (5.1)	27.7 (3.0)	0.05

IQR, interquartile range; PCR, polymerase chain reaction; CT, cycle time.

**Figure 1:**
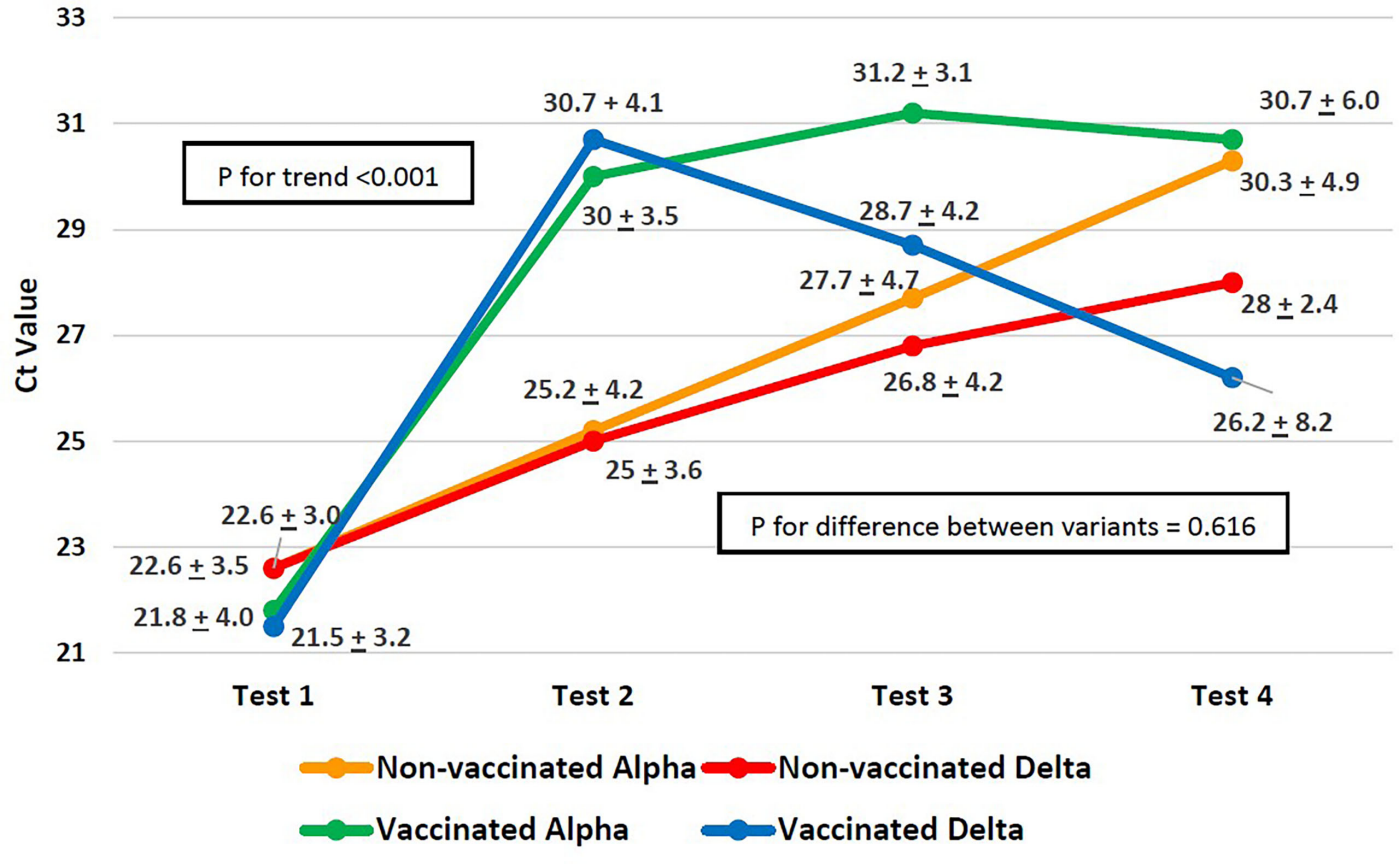
Progression of PCR cycle time (CT) values across SARS-CoV-2 variants.

The difference in the Ct value trend across the four PCR tests and the difference in the trend between the variants are shown in **Figure 1**. For both variants, regardless of the vaccination status, the increase in CT values by the fourth consecutive PCR test (**Figure 1**) was significant (p<0.001) as measured by p-value for within-subject effects using a mixed ANOVA. Although patients infected with both the variants started out with similar Ct values (**Figure 1**) at the first PCR test, in subsequent tests, the alpha variant’s Ct values increased faster, whereas the increase in Ct values for the delta variant was slow. In both vaccinated as well as unvaccinated patients, the difference in Ct values across variants, as measured by the between-subject effects using a mixed model ANOVA, was not significant (p=0.616).

**Figure 2:**
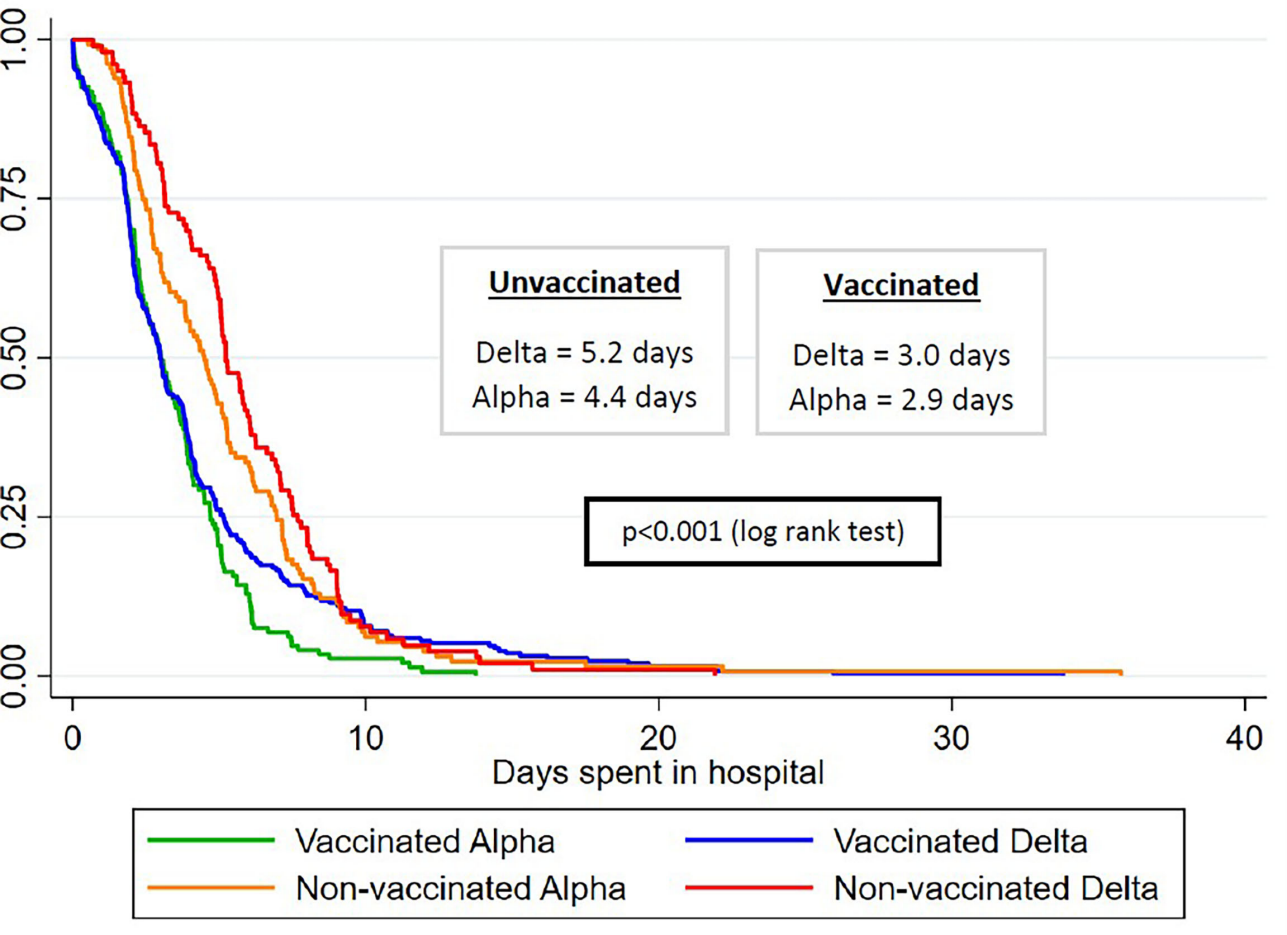
Median length of hospital stay by variant type and vaccination status.

**Figure 2** conveys the gradient in median length of hospital stay across variants between vaccinated and unvaccinated patients. Unvaccinated patients infected with the delta variant had the longest median length of stay (5.2 days), followed by unvaccinated patients infected with the alpha variant (4.4 days) and by vaccinated patients infected with the delta variant (3.0 days), and the least amount of median hospital stay was for vaccinated patients infected with the alpha variant (2.9 days). We saw 9 total deaths in our study (data not shown in the tables). Of these, two (22%) occurred in vaccinated patients, and both were infected with the alpha variant. Of the remaining 7 patients who died (78%), two were infected with the delta variant and the rest were other variants. The nonvaccinated group experienced a 1.4% mortality, which was more than thrice compared to 0.4% in the vaccinated group.

As seen in **Table 2**, after adjusting for age, sex, and vaccination status, the alpha variant’s hazards to get discharged were 1.20 (compared to the delta variant, 1.01–1.41). In other words, the delta variant was 80% more likely to stay in the hospital compared to the alpha variant (p<0.001). Unvaccinated individuals were 22% more likely to stay in the hospital compared to unvaccinated counterparts, as indicated by a hazard ratio of 1.77 (1.49–2.11) for vaccinated patients. Age appeared to marginally decrease the likelihood of discharge (HR = 0.98, p<0.001). No significant difference across sex was observed.

**Table 2:** Hazards of discharge across variant types.

Variable	HR (95% CI)	P value
**Variant**		
*Alpha*	1.20 (1.01–1.41)	0.029
*Delta*	Ref	
**Vaccination status**		
*Vaccinated*	1.77 (1.49–2.11)	<0.001
*Unvaccinated*	Ref	
**Age**		
*Age*	0.98 (0.97–0.99)	<0.001
**Sex**		
*Male*	1.17 (0.98–1.38)	0.071
*Female*	Ref	

## Discussion

The present study found the median length of stay to be higher in patients infected with the delta variant of SARS-CoV-2 compared to the alpha variant. This pattern of recovery was consistent between vaccinated as well as nonvaccinated individuals.

The average age of patients infected with the delta variant compared to the alpha variant patients suggests that younger people may be more susceptible to the delta variant. The latest data from the CDC support this finding, showing higher rates of COVID-19 hospitalizations in those aged 18–49 than previously reported during the pandemic, and higher rates than older age groups (30). This may be due to increased vaccination in older ages, resulting in comparatively fewer cases in older cohorts. Data from the American Academy of Pediatrics between July 22nd and August 5th, 2021 showed a 4% rise in COVID-19 rates in children, in addition to comprising 15% of all cases (31). In accordance with our findings, a recent article from the *British Medical Journal* reported the highest outbreaks of delta in school settings and increasing COVID rates among children in the UK (22).

A higher proportion of those infected with the alpha variant in the present study were vaccinated compared to those infected with the delta variant. It is worth noting that since the data from this study were collected between January and April of 2021, the cases of the delta variant were just beginning to rise in Bahrain, and the dominant strain was still the alpha variant (32). This could be one of the reasons we saw a higher number of breakthrough infections from the alpha variant compared to the delta variant. Recent studies, however, have found vaccine efficacy against delta to be less than that of the alpha variant. In the UK, a study found that two vaccine doses sufficiently provide protection from infection by the delta variant, but to a lesser degree than against the alpha variant (33, 34). Similarly, Lopez Bernal and coworkers have reported that although there was little difference in vaccine efficacy against the alpha and delta variant infection with 2 doses of Pfizer-BioNTech and AstraZeneca vaccines, the efficacy was still greater against the alpha variant (26). Both the AstraZeneca and Pfizer-BioNTech vaccines have been reported to be less protective against hospitalization and infection caused by the delta variant in comparison to alpha (35, 36).

The Ct trend indicated no significant difference between the variants. In contrast, a US study found a higher viral load in delta demonstrated by a lower Ct value (delta 98, alpha 562), but no difference was found between vaccinated and unvaccinated groups (37). A Chinese study recorded Ct of 24 for delta compared to Ct of 34.31 of clade19a/19b. This means that the delta variant viral load is 1,260 times higher than the first strain (23). A recent study in France found lower Ct values for delta variant infection (Ct value: 17.3; 95%CI: 15–19.7) at the time of symptoms (unadjusted for time since symptoms) compared to alpha variant infection (Ct value 19.7, 95%CI: 16.2–23) (38).

The difference between our findings and the studies could be due to time elapsed since symptom or infection onset. A study on the viral load of the alpha variant commented that low Ct values alone could not predict the detection of the strain in a population being tested at different times, especially if not early in the infection when Ct values are highest, which is around the fifth day (39). Hence, those who are asymptomatic, randomly tested, or tested after established symptoms may not demonstrate high Ct values. Our finding exemplifies a real-world scenario, in which testing takes place at different points of infection, not necessarily at the onset.

The length of stay was higher in delta compared to alpha in both the vaccinated and unvaccinated. After adjusting for age and sex, the delta variant-infected patients were 80% more likely to remain at the hospital compared to their alpha variant counterparts. In accordance with our findings, a Scottish study concluded the risk of hospital admission from delta to be twice that of those with alpha. Although those vaccinated had reduced risk of admission, being infected with the delta variant rather than the alpha variant increased their risk of hospitalization (35). In the present study, we observed a small fraction of vaccinated people infected with delta with a slower recovery (length of stay >10 days) compared to unvaccinated delta infections. Upon closer analysis of demographic data from delta infections that stayed longer than 10 days, we found that vaccinated delta variant patients with >10 days of stay were significantly older and had higher proportion of males (mean age = 42.4 years, 38% males) compared to unvaccinated patients with delta variant infection (mean age =31.8 years, 18% males), suggesting that age and male gender were likely to play a role in slower recovery within delta variant infections, as also found by Hu and coworkers (40) and Butt et al. (41).

Another factor to consider in terms of breakthrough infections in vaccinated individuals is possible immune decay, especially in those who received their vaccination earliest in January. Nations such as Bahrain and United Arab Emirates, where inactivated virus (Sinopharm) was used for inoculation in the earliest stages of the vaccination roll-out, had since updated their national guidelines for such individuals to receive booster doses (42, 43), given the possibility of waning immunity (44, 45), which is also seen in vaccinated individuals (46). Since our dataset includes participants hospitalized from January till April 2021, it is possible that some of the breakthrough infections that occurred may have been in subjects who received their doses early in January and might have experienced immune decay.

A cohort study from England over March–May 2021 comparing alpha and delta variant hospitalization risk and emergency admissions with a population of similar age (31 years on average) and findings to our study established a higher risk of hospital admission and ER attendance (within 14 days of specimen collection) with delta (24). The study also found that unvaccinated delta infections had a higher admission risk compared to unvaccinated alpha counterparts. The study did not report any difference in hospital admission risk between vaccinated delta and alpha infections, similar to the results from Sheikh et al. (35). The investigators also found delta patients to have twice the risk of hospital admission and ER use compared to alpha variant infections. Supporting our findings, the CDC Weekly Morbidity and Mortality Report from New York (47) also found vaccines to protect against hospitalization in the fully vaccinated but noted reduced efficacy against new infections even in the vaccinated when delta became the predominant strain. Prolonged hospital stay is explicable in the context of the increased admission risk and emergency facilities use caused by delta.

Despite breakthrough infections and hospital admissions, population level data on vaccinations and COVID-19 death demonstrate that the greatest protection conferred by COVID-19 vaccines worldwide is against mortality and severe disease. Nation-level COVID-19 death rates in unvaccinated individuals have been found to be anywhere between 3 and 12 times higher than that in vaccinated people in England, Northern Ireland, Singapore, and Chile (48–50). The Center for Disease Control statistics (48, 51) on COVID-19 mortality in the United States indicate that unvaccinated individuals had a death rate of 3.47 per 100,000 compared to 0.54 per 100,000 in fully vaccinated individuals (all vaccines). Similarly, data from Switzerland and Liechtenstein as reported by their Federal Office of Public Health (52), saw a death rate of 11.92 per 100,000 in unvaccinated individuals compared to 0.55 per 100,000 in vaccinated ones. Although restricted to hospitalized patients, the present study also found the mortality rate in unvaccinated subjects (1.4%) to be more than 3 times higher compared to the vaccinated group (0.4%), reiterating the global findings that COVID-19 vaccines remain highly protective against mortality and severe disease and therefore are of utmost public health significance.

### Strengths and Limitations

This paper is one of the first efforts to use real-world data from vaccination roll-out in Bahrain to look at how the recovery pattern differs between alpha and delta variants of concern of SARS-CoV-2. One of the limitations of this body of work is that we did not have data on immune responses to see how immune decay in individuals vaccinated early on would have played a role in breakthrough infections.

## Conclusion

In conclusion, the present study found patients infected with the delta variant of SARS-CoV-2 to have longer recovery periods as indicated by length of stay and pattern of PCR test CT values, within both vaccinated and unvaccinated individuals. In light of these findings, relaxation of preventative public health measures such as mask mandates needs to be evaluated in the context of proportion of SARS-CoV-2 infections that are the delta variant.

## Data Availability Statement

The raw data supporting the conclusions of this article will be made available by the authors, without undue reservation.

## Ethics Statement

The studies involving human participants were reviewed and approved by the National COVID-19 Research Committee in Bahrain; Approval Code: CRT-COVID2021-148. Written informed consent for participation was not required for this study in accordance with the national legislation and the institutional requirements.

## Author Contributions

NK analyzed and interpreted the data, and wrote and reviewed the manuscript. SQ wrote and reviewed the manuscript. AA oversaw the data collection and reviewed the manuscript. MA was responsible for conception, design, and overall responsibility of the study, as well as wrote and reviewed the manuscript. All authors contributed to the article and approved the submitted version.

## Funding

Financial support for open access publication was received from RCSI -Bahrain, project number: 172/22-Aug-2021.

## Conflict of Interest

The authors declare that the research was conducted in the absence of any commercial or financial relationships that could be construed as a potential conflict of interest.

## Publisher’s Note

All claims expressed in this article are solely those of the authors and do not necessarily represent those of their affiliated organizations, or those of the publisher, the editors and the reviewers. Any product that may be evaluated in this article, or claim that may be made by its manufacturer, is not guaranteed or endorsed by the publisher.
